# A Robust Adaptive Filtering Framework for Smartphone GNSS/PDR-Integrated Positioning

**DOI:** 10.3390/mi17030353

**Published:** 2026-03-13

**Authors:** Jijun Geng, Chao Liu, Chao Song, Chao Chen, Yang Xu, Qianxia Li, Peng Jiang, Congcong Wu

**Affiliations:** 1 Key Laboratory of Aviation-Aerospace-Ground Cooperative Monitoring and Early Warning of Coal Mining-Induced Disasters of Anhui Higher Education Institutes, Anhui University of Science and Technology, Huainan 232001, China; 2 Key Laboratory of Geoghysical Exploration Equipment, Ministry of Education, Jilin University, Changchun 130026, China; 3 Key Laboratory of CAD&CG, Zhejiang University, Hangzhou 310058, China; 4 Key Laboratory of Space Photoelectric Detection and Perception, Nanjing University of Aeronautics and Astronautics, Ministry of Industry and Information Technology, No.29 Jiangjun Street, Jiangning District, Nanjing 211106, China; 5 State Key Laboratory for Safe Mining of Deep Coal Resources and Environment Protection, Anhui University of Science and Technology, Huainan 232001, China; 6 Anhui Provincial Key Laboratory of Joint Construction Disciplines for Urban Real Scene 3D and Intelligent Security Monitoring, Huainan 232001, China; 7 Xi’an Tianmu Surveying, Mapping and Geoinformation Co., Ltd., Xi’an 710030, China; 8 School of Geography and Planning, Sun Yat-sen University, 135 # Xingangxi Road, Guangzhou 510275, China; 9 Surveying and Mapping Institute Lands and Resource Department Guangdong Province, Guangzhou 510500, China; 10 Guangzhou South Surveying & Mapping Technology Co., Ltd., Guangzhou 510500, China; 11 School of Graduate, Anhui University of Science and Technology, Huainan 232001, China

**Keywords:** complex environments, smartphone positioning, multi-sensor fusion, pedestrian dead reckoning, robust adaptive filtering

## Abstract

Accurate and continuous outdoor pedestrian positioning using smartphones remains challenging in complex environments like urban canyons, where Global Navigation Satellite System (GNSS) signals are frequently degraded or blocked, and Pedestrian Dead Reckoning (PDR) suffers from cumulative errors. To address this, this paper proposes a novel fusion method based on a Robust Adaptive Cubature Kalman Filter (RACKF). The core of our approach is a two-stage filtering architecture: the first stage employs a quaternion-based RACKF to optimally fuse gyroscope and magnetometer data for robust heading estimation; the second stage performs the core fusion of GNSS observations with an enhanced 3D PDR solution. Key innovations include an adaptive noise estimation strategy combining fading and limited memory weighting, a robust M-estimator-based mechanism to suppress outliers, and the integration of differential barometric height measurements. Experimental results demonstrate that the proposed method achieves a horizontal positioning accuracy of 3.28 m (RMSE), outperforming standalone GNSS and improving 3D PDR by 25.97% and 10.39%, respectively. This work provides a practical, infrastructure-free solution for robust smartphone-based outdoor navigation.

## 1. Introduction

Smartphone-based PDR achieves relative position updates through step frequency detection, step length estimation, and heading calculation [1]. However, a primary limitation of PDR is error accumulation, stemming from the inherent limitations of smartphone sensors: gyroscope bias instability and random walk lead to unbounded heading drift after integration; the magnetometer is susceptible to local magnetic field distortions, reducing its reliability in dynamic environments; although double integration of accelerometer data is theoretically optimal for direct step length estimation, bias and noise cause displacement errors to increase cubically [2,3,4,5]. Furthermore, unlike foot-mounted devices, the diverse carrying modes of smartphones—such as in pockets, handheld, or in backpacks—cannot guarantee that the sensor axes remain continuously parallel to the gait direction. This results in step length model mismatch and heading randomness, which become the primary error sources for smartphone-based PDR and significantly exacerbate long-term positioning drift.

For GNSS positioning in smartphones, challenges are equally significant. While nominal accuracy in open sky is approximately 10 m, performance severely degrades in urban canyons due to signal multipath, non-line-of-sight reception, and frequent outages [6,7,8]. The low-cost antennas and chipsets in consumer smartphones further exacerbate these issues, leading to unstable pseudorange measurements and unreliable position fixes [9,10]. Although advanced techniques like carrier-phase smoothing and multi-constellation support are being explored for mobile devices [11,12], they often require external hardware or lack robustness under severe signal dynamics. Therefore, relying solely on GNSS is insufficient for continuous and reliable pedestrian navigation in complex urban settings [13,14,15].

To overcome the limitations of individual sensors, multi-sensor fusion has become an inevitable trend [16,17,18,19]. Fusion strategies can be categorized as loosely coupled or tightly coupled based on their coupling strength: the former independently processes GNSS and PDR outputs before fusing them at the filter level, offering flexibility but failing to exploit raw observation information; the latter directly fuses GNSS pseudorange/carrier phase with Inertial Measurement Unit (IMU) data, offering higher accuracy but imposing stringent requirements on computational resources [20,21,22,23]. The Kalman Filter (KF) and its variants have served as the mainstream fusion framework, evolving from the Extended Kalman Filter (EKF) to the Unscented Kalman Filter (UKF) and further to the Cubature Kalman Filter (CKF) [24,25,26,27,28]. The EKF is prone to divergence in highly nonlinear systems due to linearization errors; the UKF employs Sigma point transformation but suffers from numerical instability issues caused by negative weights; the CKF, relying on the third-degree spherical–radial cubature rule, strictly approximates Gaussian weighted integrals using 2n equally weighted cubature points, offering both numerical stability and computational efficiency [29,30,31,32]. However, these standard filters often assume fixed and known noise statistics, which is unrealistic in dynamic smartphone environments where sensor noise and GNSS error characteristics are time-varying and occasionally contain outliers [33,34,35].

This paper proposes a Robust Adaptive Cubature Kalman Filter (RACKF) framework for tightly coupled GNSS/PDR fusion. The main contributions are threefold: (1) We propose a two-stage RACKF architecture—the first-stage filter optimizes heading estimation, and the second-stage filter simultaneously resolves planar position and elevation. By introducing fading memory and finite memory weighting mechanisms to dynamically adjust system noise, combined with an adaptive factor based on prediction residuals and a robust factor based on maximum likelihood estimation, it effectively suppresses time-varying noise from smartphone sensors and interference from GNSS abnormal observations, overcoming the excessive dependence of traditional filters on prior noise statistics. (2) We improve the heading angle selection strategy for smartphone-based PDR by adopting a single-step heading averaging method to reduce the impact of random handheld posture, and establish a nonlinear step length model using the maximum–minimum acceleration within a single step as the feature, which can adapt to different gaits without requiring individual parameter calibration. (3) We innovatively introduce joint constraints of differential barometric height measurement and step frequency detection, incorporating elevation estimation into a unified filtering framework to achieve simultaneous robust resolution of planar coordinates and vertical height.

The proposed scheme relies entirely on built-in smartphone sensors, requiring no pre-established fingerprint databases, physical maps, or additional infrastructure, forming a lightweight, ready-to-use 3D positioning model. Through the tight coupling of multi-source information via RACKF, it effectively suppresses long-term drift of MEMS sensors and short-term jumps in GNSS, significantly improving positioning continuity and robustness in complex scenarios such as urban canyons. This method balances algorithmic accuracy with computational efficiency on mobile platforms, providing a cost-effective, highly adaptable, and practical solution for smartphone-based outdoor pedestrian navigation.

## 2. Materials and Methods

### 2.1. Integration Architecture

**Figure 1:**
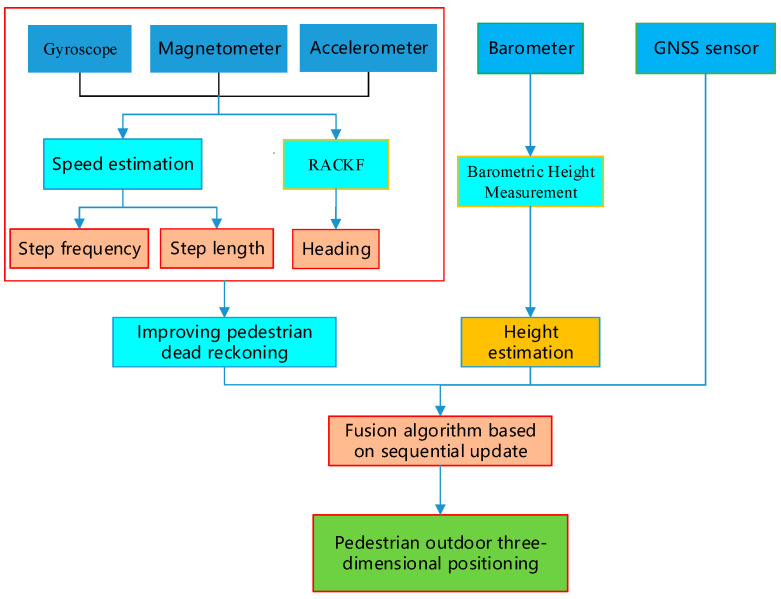
Proposed joint filtering integration scheme.

This study focuses on outdoor pedestrian positioning using low-cost, lightweight smartphones equipped with multiple sensors, through the fusion of multi-source signals and the coordination of multiple methods. Based on an in-depth analysis of the characteristics and mechanisms of positioning influencing factors, a three-dimensional pedestrian dead reckoning (PDR) model is constructed to enhance the adaptability and robustness of the algorithm, improve the accuracy and stability of heading estimation, and effectively suppress the accumulation of positioning errors. Furthermore, by integrating GNSS positioning technology, this study aims to achieve continuous and reliable 3D positioning services in complex outdoor environments, thereby improving the precision, availability, and reliability of pedestrian positioning, as well as its theoretical and practical value. This research utilizes built-in smartphone sensors such as gyroscopes, magnetometers, and accelerometers to estimate pedestrian step frequency, step length, and heading in real time. Planar coordinates are calculated based on the PDR algorithm. Meanwhile, elevation information is obtained using barometer data, and a Robust Adaptive Cubature Kalman Filter (RACKF) algorithm is employed to fuse and optimize the 3D coordinates from PDR with GNSS positioning results, ultimately outputting high-confidence 3D pedestrian locations. This method effectively enhances the environmental adaptability and output stability of the positioning system, providing a feasible technical solution for outdoor pedestrian positioning. The overall technical workflow is illustrated in Figure 1.

### 2.2. Pedestrian Outdoor Positioning Model

When location-based services are applied in outdoor environments, they require access to and rely on accurate positioning and navigation information that is continuously available in both time and space [36,37,38,39]. More specifically, such services must enable continuous estimation of pedestrian locations during transitions across complex outdoor spatial environments to ensure accuracy, availability, and continuity.

#### 2.2.1. GNSS Observation and Stochastic Model

The basic pseudorange observation equation describes the relationship between the measured pseudorange and the geometric distance, clock offsets, and residual errors:

(1) $$P=\rho +c\cdot (t_{r}-t_{s})+\varepsilon$$

where *P* is the measured pseudorange, *ρ* is the geometric range between the satellite and receiver (m), *c* is the speed of light, *t_r_* and *t_s_* are the receiver and satellite clock offsets, and *ε* represents the code noise.

A more comprehensive form, including major error sources, can be expressed as:

(2) $$P=\rho +c\cdot (t_{r}-t_{s})+I+T+M+\varepsilon_{P}$$

where *I* and *T* are the ionospheric and tropospheric delays, *M* represents the multipath effect (m), and *ε_P_* is the receiver noise.

For the purpose of recursive state estimation, the pseudorange observation equation is often linearized around the predicted state. The linearized pseudorange residual is given by:

(3) ΔP=eΔx+c·Δt+ε

where Δ*P* is the difference between the measured and computed pseudorange, e is the line-of-sight unit vector from the receiver to the satellite, Δ*x* is the position error vector, Δ*t* is the receiver clock offset error, and *ε* is the composite measurement noise.

To mitigate atmospheric errors, standard correction models are applied. The ionospheric delay *I* is corrected using the Klobuchar model with parameters broadcast in the navigation message [13], and the tropospheric delay *T* is corrected using the Saastamoinen model with standard meteorological parameters and the Niell mapping function [14]. After applying these corrections, the corrected pseudorange observation can be written as:

(4) $$P_{\mathrm{corr}}=\rho +c\cdot (t_{r}-t_{s})+\varepsilon^{\prime }$$

where *ε*′ denotes the combined residual error after atmospheric corrections.

Given that the quality of GNSS measurements strongly depends on the satellite elevation angle, an elevation-dependent stochastic model is adopted to weight the observations. The variance of the pseudorange measurement noise is modeled as:

(5) $$\delta^{2}{=a}^{2}+b^{2}/\sin^{2}(el)$$

where *el* is the satellite elevation angle, and a and b are empirical constants that characterize the base noise level and the elevation-dependent error component, respectively [13]. This scheme assigns lower weights to low-elevation satellites, which are more susceptible to multipath and atmospheric errors.

#### 2.2.2. Integrated Positioning Model Based on GNSS and Improved 3D PDR

Existing models predominantly adopt two approaches: GNSS-based positioning and PDR-based positioning [40]. The fundamental idea behind such positioning algorithms is as follows: in GNSS mode, if satellite positioning yields a coordinate solution, that coordinate is output as the final positioning result. In pedestrian dead reckoning (PDR) mode, the user’s relative positioning result—obtained by calculating step length and heading—is used as the final positioning output. However, in complex environments such as urban canyons, GNSS signals are often blocked by tall buildings and structures. Although GNSS receivers may still obtain coordinate fixes under such conditions, the positioning error can be substantial. If the final positioning result relies solely on GNSS data, positioning accuracy will be poor. Conversely, if PDR is used as an alternative when GNSS signals are weak, the accuracy of the positioning result cannot be guaranteed over longer distances due to the inherent error accumulation in PDR.

Under these circumstances, an optimal approach is to combine the positioning results from GNSS with the relatively high short-term accuracy of PDR data. By integrating GNSS single-point positioning with PDR positioning data, better overall performance can be achieved. Therefore, in multi-sensor fusion-based pedestrian positioning methods, a GNSS and an improved 3D PDR-integrated positioning model is adopted. The “improved 3D PDR” specifically includes: (1) a single-step heading averaging method to mitigate random handheld posture noise; (2) a nonlinear step length model based on the maximum and minimum acceleration within a gait cycle; and (3) the introduction of joint constraints from differential barometry and step frequency for elevation estimation. When GNSS data is available, or in environments like urban canyons where GNSS signals are mostly poor but occasionally provide reliable positioning fixes, the improved 3D PDR is fused with GNSS single-point positioning using robust adaptive filtering to optimize the positioning data, mitigate error accumulation, and enhance positioning accuracy.

### 2.3. Attitude and Heading Determination Based on a Robust Adaptive Cubature Kalman Filter Algorithm

The heading and attitude angles are calculated using the coordinate transformation matrix $C_{b}^{n}$, which transforms vectors from the body (b) frame to the navigation (n) frame [18]:

(6) $$C_{b}^{n}=\left[\begin{array}{ccc} q_{0}^{2}+q_{1}^{2}-q_{2}^{2}-q_{3}^{2} & 2\left(q_{1}q_{2}-q_{0}q_{3}\right) & 2\left(q_{1}q_{3}+q_{0}q_{2}\right)\\ 2\left(q_{1}q_{2}+q_{0}q_{3}\right) & q_{0}^{2}-q_{1}^{2}+q_{2}^{2}-q_{3}^{2} & 2\left(q_{2}q_{3}-q_{0}q_{1}\right)\\ 2\left(q_{1}q_{3}-q_{0}q_{2}\right) & 2\left(q_{2}q_{3}+q_{0}q_{1}\right) & q_{0}^{2}-q_{1}^{2}-q_{2}^{2}+q_{3}^{2} \end{array}\right]$$

In this work, the navigation frame (n-frame) is defined with an East-North-Up (ENU) orientation. The inverse transformation matrix, from the n-frame to the b-frame, is given by $C_{n}^{b}$ and can be expressed as follows [3,7]:

(7) $$C_{n}^{b}=\left[\begin{array}{ccc} cos\varphi cos\psi +\;sin\varphi sin\psi sin\theta & -cos\varphi sin\psi +\;sin\varphi cos\psi sin\theta & -\sin\varphi \cos\theta \\ sin\psi cos\theta & cos\psi cos\theta & sin\theta \\ sin\varphi \;cos\psi -cos\varphi sin\psi sin\theta & -sin\varphi sin\psi -\;cos\varphi cos\psi sin\theta & \cos\varphi \cos\theta \end{array}\right]$$

where ψ is the yaw angle; φ is the pitch angle; θ is roll angle.

Based on Equations (6) and (7), the Euler angles can be represented using a unit quaternion as follows:

(8) $$\begin{array}{c} \theta =\arcsin\left[2\left(q_{2}q_{3}+q_{0}q_{1}\right)\right]\\ \varphi =\arctan\left[-\frac{2\left(q_{1}q_{3}-q_{0}q_{2}\right)}{1-{q_{1}}^{2}-{q_{2}}^{2}+{q_{3}}^{2}}\right]\\ \psi_{m}=\arctan\left[\frac{2\left(q_{1}q_{2}-q_{0}q_{3}\right)}{1-{q_{1}}^{2}+{q_{2}}^{2}-{q_{3}}^{2}}\right] \end{array}$$

The true heading ψ is obtained by correcting the yaw angle $\psi_{m}$ with the local magnetic declination D:

(9) $$\psi =\psi_{m}+D$$

where D is the local declination angle.

The attitude quaternion $\dot{q}$ describes the full orientation. The incremental change in the quaternion, representing the attitude and heading update from the previous state, is governed by the kinematic equation:

(10) $$\dot{q}=\frac{1}{2}q⊗w$$

where w is the angular rate vector measured by the gyroscope.

The discrete-time process and observation models for the filter are:

(11) $$\begin{array}{l} X_{k}=F_{k-1}X_{k-1}+w_{k-1}\\ z_{k}=h\left(X_{k}\right)+v_{k} \end{array}$$

where $X_{k}={\left[q0\;q1\;q2\;q3\right]}^{T}$, $F_{k-1}=I\times \cos(\vartheta /2)+A\times dt\times \sin(\vartheta /2)/\vartheta$, $z_{k}={\left[a_{x}\;a_{y}\;a_{z}\;m_{x}\;m_{y}\;m_{z}\right]}^{T}$, $h\left(X_{k}\right)=\left(\begin{array}{c} 2(q_{1}q_{3}-q_{0}q_{2})\\ 2(q_{2}q_{3}+q_{0}q_{1})\\ q_{0}^{2}-q_{1}^{2}-q_{2}^{2}+q_{3}^{2}\\ 2(q_{1}q_{2}+q_{0}q_{3})m_{N}+2(q_{1}q_{3}-q_{0}q_{2})m_{U}\\ (q_{0}^{2}-q_{1}^{2}+q_{2}^{2}-q_{3}^{2})m_{N}+2(q_{2}q_{3}+q_{0}q_{1})m_{U}\\ 2(q_{2}q_{3}-q_{0}q_{1})m_{N}+(q_{0}^{2}-q_{1}^{2}-q_{2}^{2}+q_{3}^{2})m_{U} \end{array}\right)$, $w_{k-1}$ and $v_{k}$ are the noises.

**Time Update**

Assume the posterior density at time k − 1 is known. First, perform Cholesky factorization:

(12) $$P_{k-1|k-1}=S_{k-1|k-1}S_{k-1|k-1}^{T}$$

Then, calculate the cubature points $X_{i,k-1|k-1}$ (for i = 1, 2, …, m, where m = 2n):

(13) $$X_{i,k-1|k-1}=S_{k-1|k-1}\xi_{i}+{\hat{x}}_{k-1|k-1}$$

where $\xi_{i}$ the basic cubature points. Propagate these points through the state equation $X_{i,k|k-1}^{*}$:

(14) $$X_{i,k|k-1}^{*}=f\left(X_{i,k-1|k-1},u_{k-1}\right)$$

where f(.) is the known function; $u_{k-1}$ is the system noise.

The state prediction ${\hat{x}}_{k|k-1}$ and state prediction covariance $P_{k|k-1}$ are then estimated as:

(15) $${\hat{x}}_{k|k-1}=\frac{1}{m}\overset{m}{\underset{i=1}{\sum }}X_{i,k|k-1}^{*}$$

(16) $$P_{k|k-1}=\frac{1}{m}\overset{m}{\underset{i=1}{\sum }}X_{i,k|k-1}^{*}X_{i,k|k-1}^{*T}-{\hat{x}}_{k|k-1}{\hat{x}}_{k|k-1}^{T}+Q_{k-1}$$

To address time-varying process noise, an adaptive strategy combining Fading Memory Weighting (FMW) and Limited Memory Weighting (LMW) methods is employed. The FMW-based covariance ${\hat{Q}}_{k}$ is calculated as [15]:

(17) $${\hat{Q}}_{k}=\left(1-d_{k}\right){\hat{Q}}_{k-1}+d_{k}[W_{k}\varepsilon_{k}\varepsilon_{k}^{T}W_{k}^{T}+P_{k|k}-(\frac{1}{2n}\overset{m}{\underset{i=1}{\sum }}X_{i,k|k-1}^{*}X_{i,k|k-1}^{*T}-{\hat{x}}_{k|k-1}{\hat{x}}_{k|k-1}^{T})]$$

where $d_{k}=(1-b)/(1-b^{k+1})$, b is the forgetting factor and 0.95<b<0.99; $\varepsilon_{k}$ is the filter innovation; $\varepsilon_{k}=z_{k}-{\hat{z}}_{k|k-1}$; $W_{k}$ is the innovation vector; and $P_{k|k}$ is the corresponding error covariance.

The LMW-based covariance ${\hat{Q}}_{k}$, using a memory window of length w, is expressed as [18]:

(18) $${\hat{Q}}_{k}=b{\hat{Q}}_{k-1}+d_{w}[W_{k}\varepsilon_{k}\varepsilon_{k}^{T}W_{k}^{T}+P_{k|k}-(\frac{1}{2n}\overset{2n}{\underset{i=1}{\sum }}X_{i,k|k-1}^{*}X_{i,k|k-1}^{*T}-{\hat{x}}_{k|k-1}{\hat{x}}_{k|k-1}^{T})]+d_{w}b^{w}{\hat{Q}}_{k-w}$$

where ${\hat{Q}}_{k-w}=W_{k-w}\varepsilon_{k-w}\varepsilon_{k-w}^{T}W_{k-w}^{T}+P_{k-w|k-w}-(\frac{1}{2n}\overset{2n}{\underset{i=1}{\sum }}X_{i,k-w|k-w-1}^{*}X_{i,k-w|k-w-1}^{*T}-{\hat{x}}_{k-w|k-w-1}{\hat{x}}_{k-w|k-w-1}^{T})$; $d_{w}=\left(1-b\right)/\left(1-b^{w}\right)$; b is the forgetting factor. In this paper, the FMW method initializes Qk from the start until time k − w, after which the LMW method takes over from time k − w + 1. This hybrid approach enhances the accuracy of noise estimation.

**Measurement Update**

Factorize the predicted covariance:

(19) $$P_{k|k-1}=S_{k|k-1}S_{k|k-1}^{T}$$

Calculate the cubature points for the measurement update $X_{i,k|k-1}$:

(20) $$X_{i,k|k-1}=S_{k|k-1}\xi_{i}+{\hat{x}}_{k|k-1}$$

Propagate these points through the measurement equation $Z_{i,k|k-1}$:

(21) $$Z_{i,k|k-1}=h\left(X_{i,k|k-1},v_{k}\right)$$

where $h\left(.\right)$ is a known function, $v_{k}$ is the measurement noise.

The predicted measurement ${\hat{z}}_{K|K-1}$ and the innovation covariance matrix $P_{zz,k|k-1}$:

(22) $${\hat{z}}_{K|K-1}=\frac{1}{m}\overset{m}{\underset{I=1}{\sum }}Z_{i,k|k-1}$$

(23) $$P_{zz,k|k-1}=\frac{1}{m}\overset{m}{\underset{I=1}{\sum }}Z_{i,k|k-1}Z_{i,k|k-1}^{T}-{\hat{z}}_{k|k-1}{\hat{z}}_{k|k-1}^{T}+R_{k}$$

where $R_{k}$ is the measurement noise covariance.

An adaptive factor α_k_ is introduced to mitigate the impact of dynamic model errors and abnormal disturbances, as pedestrian motion is often irregular and susceptible to interference [24,25,26,27,28,29]. This factor, based on predicted state discrepancy statistics, is defined using a two-segment function [7]:

(24) $$\partial_{k}=\left\{\begin{array}{cc} 1, & \Delta {\tilde{X}}_{k}\leq c0\\ \frac{c0}{\Delta {\tilde{X}}_{k}}, & \Delta {\tilde{X}}_{k}>c0 \end{array}\right.$$

where c0 is a tunable constant; $\Delta {\tilde{V}}_{k}$ is the statistic of the predicted state discrepancy, defined as: $\Delta {\tilde{X}}_{k}={\left[\left\|{\tilde{X}}_{k}-{\hat{x}}_{k|k-1}\right\|/tr\left(\mathrm{cov}(\varepsilon_{k},{\varepsilon_{k}}^{T}\right)\right]}^{\frac{1}{2}}$; ${\tilde{X}}_{k}$ is a least-square estimator of the state; and *tr*(·) stands for the trace of a matrix.

The innovation covariance matrix $P_{zz,k|k-1}^{*}$ is then adaptively corrected as [3]:

(25) $$P_{zz,k|k-1}^{*}=\frac{1}{m}\overset{m}{\underset{I=1}{\sum }}Z_{i,k|k-1}Z_{i,k|k-1}^{T}-{\hat{z}}_{k|k-1}{\hat{z}}_{k|k-1}^{T}+\partial_{k}{\bar{R}}_{k}$$

To robustly handle measurement outliers, an M-estimator-based equivalent weight matrix ${\bar{R}}_{k}$ is applied. This paper employs Huber’s method [30]. The diagonal ${\bar{r}}_{k_{ii}}$ and non-diagonal ${\bar{r}}_{k_{ij}}$ elements of ${\bar{R}}_{k}$ are corrected as [7]:

(26) $${\bar{r}}_{k_{ii}}=\left\{\begin{array}{cc} \frac{1}{\sigma_{ii}}, & \left|r_{k_{i}}^{\prime }\right|\leq c\\ \frac{c}{\left|r_{k_{i}}^{\prime }\right|}\cdot \frac{1}{\sigma_{ii}}, & \left|r_{k_{i}}^{\prime }\right|>c \end{array}\right.$$

(27) $${\bar{r}}_{k_{ij}}=\left\{\begin{array}{cc} \frac{1}{\sigma_{ij}}, & \left|r_{k_{i}}^{\prime }\right|\leq c\;and\;\left|r_{k_{j}}^{\prime }\right|\leq c\\ \frac{c}{\max\left\{\left|r_{k_{i}}^{\prime }\right|,\left|r_{k_{j}}^{\prime }\right|\right\}}\cdot \frac{1}{\sigma_{i,j}}, & \left|r_{k_{i}}^{\prime }\right|>c\;or\;\left|r_{k_{j}}^{\prime }\right|>c \end{array}\right.$$

where $\sigma_{ii}$ and $\sigma_{ij}$ are elements of ***R_k_***. *c* is a constant typically within [1.3, 2.0]. $r_{k_{i}}^{\prime }$ is the standardized residual:

(28) $$\left|r_{k_{i}}^{\prime }\right|=\left|\frac{r_{k_{i}}}{\sigma_{r_{k_{i}}}}\right|$$

where $r_{k_{i}}$ is the residual of the measurement $z_{k_{i}}$; $\sigma_{r_{k_{i}}}$ is the mean deviation of $r_{k_{i}}$.

Finally, the cross-covariance matrix, Kalman gain, and the state and covariance updates are computed:

(29) $$P_{xz,k|k-1}=\frac{1}{m}\overset{m}{\underset{I=1}{\sum }}X_{i,k|k-1}Z_{i,k|k-1}^{T}-{\hat{x}}_{k|k-1}z_{k|k-1}^{T}$$

(30) $$W_{k}=P_{zz,k|k-1}P_{xz,k|k-1}^{-1}$$

(31) $${\hat{x}}_{k|k}={\hat{x}}_{k|k-1}+W_{k}\left(z_{k}-{\hat{z}}_{k|k-1}\right)$$

(32) $$P_{k|k}=P_{k|k-1}-W_{k}P_{zz,k|k-1}W_{k}^{-1}$$

### 2.4. Speed Estimation

Speed estimation in pedestrian dead reckoning primarily involves two core components: step frequency detection and step length estimation [3]. The purpose of step frequency detection is to accurately identify the start and cycle of individual gait strides from continuous sensor data. This segmentation into independent gait cycles forms the basis for subsequent calculations of step length and heading. Current step detection algorithms based on MEMS sensors mainly include methods such as peak detection, threshold setting, zero-velocity update (ZUPT), autocorrelation, and finite-state machine (FSM) [31]. Considering the periodic characteristics exhibited by acceleration signals during walking, this paper adopts the peak detection method. This method determines gait events by analyzing the periodic peaks of the resultant acceleration within a fixed time window. The resultant acceleration used for detection is calculated from the measurements of a triaxial accelerometer, and its numerical fluctuations can effectively reflect walking patterns.

Research indicates that step length is related to various factors such as a pedestrian’s acceleration, height, and stride characteristics, with results from different estimation methods showing minor differences [7]. This paper employs a nonlinear step length estimation model. This model uses the maximum and minimum acceleration within a single gait cycle as characteristic quantities. The calculation formula is as follows [32]:

(33) $$L_{k}=S\times \sqrt[{4}]{\left(a_{\max}-a_{\min}\right)}$$

where L_k_ is the estimated length; $a_{\max}$ and $a_{\min}$ are the maximum and minimum values of the resultant acceleration within that gait cycle, respectively; and S is a personalized parameter that requires calibration for different pedestrians.

### 2.5. RAKF-Based Dynamic Estimation Model for Gait Parameters

To enhance the estimation accuracy and robustness of step length and heading across consecutive gait cycles, this section establishes a Robust Adaptive Kalman Filter (RAKF) framework. This framework leverages the strong correlation between adjacent gait parameters while incorporating an adaptive mechanism to counteract the effects of dynamic model uncertainty and sensor measurement anomalies.

During normal walking, step length and heading typically exhibit continuity and gradual variation between consecutive steps. This characteristic allows the estimated values from the previous step to serve as reliable priors for the current step’s parameters. Based on this, the state vector is defined as Xk, representing the step length and heading at the k step, respectively. Integrating gait dynamic priors with real-time sensor measurements, the following state-space model is formulated:

(34) $$\begin{array}{l} X_{k}=F_{k-1}X_{k-1}+w_{k-1}\\ z_{k}=H_{k}X_{k}+v_{k} \end{array}$$

where $X_{k}={\left[\psi \;L\right]}^{T}$, $F_{k-1}=\left[\begin{array}{cc} 1 & 0\\ 0 & 1 \end{array}\right]$ is the state transition matrix encoding the evolutionary relationship between step parameters, $z_{k}=\left[\begin{array}{l} \psi \\ L \end{array}\right]$, $H_{k}=\left[\begin{array}{cc} 1 & 0\\ 0 & 1 \end{array}\right]$ is the measurement matrix, $w_{k-1}$ and $v_{k}$ are the process noise and measurement noise, respectively, ψ is the heading, and L is the step length.

The adopted RAKF algorithm recursively solves for the optimal state estimate through two steps: prediction and update. It enhances robustness by utilizing an adaptive factor $\partial_{k}$ and an equivalent weight matrix ${\bar{R}}_{k}$.

The adaptive factor $\partial_{k}$ is constructed based on the prediction residual. Given that pedestrian motion is prone to disturbances and is challenging to model precisely, conventional filtering is susceptible to model errors. The factor $\partial_{k}$ follows a two-segment function:

(35) $$\partial_{k}=\left\{\begin{array}{cc} 1, & \Delta {\tilde{X}}_{k}\leq c0\\ \frac{c0}{\Delta {\tilde{X}}_{k}}, & \Delta {\tilde{X}}_{k}>c0 \end{array}\right.$$

where c0 is an adjustable constant and $\Delta {\tilde{X}}_{k}$ is the statistic used to judge state model errors, calculated as:

(36) $$\Delta {\tilde{X}}_{k}={\left[\left\|{\tilde{X}}_{k}-{\hat{X}}_{k|k-1}\right\|/tr\left({\hat{P}}_{k|k-1}\right)\right]}^{\frac{1}{2}}$$

where tr(·) denotes the trace of a matrix, ${\tilde{X}}_{k}$ is the weighted least squares estimate of the state:

(37) $${\tilde{X}}_{k}={\left(A_{k}^{T}P_{k}A_{k}\right)}^{-1}A_{k}^{T}P_{k}Z_{k}$$

where $P_{k}$ is the weight matrix.

To mitigate measurement outliers (gross errors), a robust M-estimation-based approach is employed to compute the equivalent weight matrix ${\bar{R}}_{k}$. Its elements are downweighted according to the standardized residual:

(38) $${\bar{r}}_{k_{ii}}=\left\{\begin{array}{cc} \frac{1}{\sigma_{ii}}, & \left|r_{k_{i}}^{\prime }\right|\leq c\\ \frac{c}{\left|r_{k_{i}}^{\prime }\right|}\cdot \frac{1}{\sigma_{ii}}, & \left|r_{k_{i}}^{\prime }\right|>c \end{array}\right.$$

(39) $${\bar{r}}_{k_{ij}}=\left\{\begin{array}{cc} \frac{1}{\sigma_{ij}}, & \left|r_{k_{i}}^{\prime }\right|\leq c\;and\;\left|r_{k_{j}}^{\prime }\right|\leq c\\ \frac{c}{\max\left\{\left|r_{k_{i}}^{\prime }\right|,\left|r_{k_{j}}^{\prime }\right|\right\}}\cdot \frac{1}{\sigma_{i,j}}, & \left|r_{k_{i}}^{\prime }\right|>c\;or\;\left|r_{k_{j}}^{\prime }\right|>c \end{array}\right.$$

where c is a constant usually chosen within [1.3, 2.0]; ${\bar{r}}_{k_{ii}}$ and ${\bar{r}}_{k_{ij}}$ are elements of the original measurement noise covariance matrix **R***_k_*.

### 2.6. Height Estimation Based on a Barometer

In complex outdoor environments—such as urban canyons, dense forests, or areas near tunnels—Global Navigation Satellite System (GNSS) signals are often weakened or interrupted, posing challenges to traditional altitude measurement methods that rely on satellite signals. Compared to laser ranging, which is susceptible to interference from complex terrain, and inertial sensors, which accumulate significant integration errors and are unsuitable for prolonged standalone operation, barometers offer unique advantages in weak GNSS environments due to their simple design, low power consumption, and ability to provide absolute altitude references without depending on external signals.

The fundamental principle of barometric altimetry is based on the physical relationship where atmospheric pressure decreases approximately exponentially with increasing altitude. By measuring the atmospheric pressure at a given point, it can be converted to an altitude relative to standard atmospheric pressure. To suppress barometric sensor noise and high-frequency disturbances, this study applies mean filtering to the original pressure observation sequence, obtaining a smoothed average pressure value [33].

To mitigate the impact of common atmospheric disturbances—such as the passage of large-scale weather systems and diurnal temperature variations—on single-point barometric altimetry and to improve measurement reliability and accuracy in complex outdoor environments, this paper employs the differential barometric altimetry technique. This method involves establishing a fixed reference station at a known location with a precisely surveyed altitude to monitor the local absolute pressure in real time. A mobile user synchronously measures the pressure at the point of interest. By calculating the pressure difference between the two, common-mode atmospheric pressure variations can be significantly canceled out. The relative height difference ΔH of the mobile point with respect to the reference station is calculated using the following differential model based on the hydrostatic equation:

(40) $$\Delta H=H_{k}-H_{0}=4946.55\times [({\bar{p}}_{k}^{0.1902631}-{\bar{p}}_{i}^{0.1902631})]$$

The absolute altitude of the mobile user H is obtained by H = H0 + ΔH. This method unifies the altitude datum to the known reference station, not only attenuating common atmospheric disturbances but also localizing the influence of complex environmental variables. Consequently, it provides an autonomous, continuous, and relatively high-precision elevation measurement solution for complex outdoor areas where GNSS signals are limited or unavailable.

### 2.7. Fusion Method

In complex environments such as urban canyons, indoors, or areas with high signal obstruction, the GNSS sensor module in smartphones often cannot receive sufficiently strong satellite signals due to blocking, multipath effects, etc., resulting in weak or lost signals. To address such scenarios, this study proposes a positioning algorithm that integrates GNSS with the improved 3D pedestrian dead reckoning (PDR) method. By fully leveraging multi-sensor information and introducing the robust adaptive Kalman filter (RAKF) framework for data fusion, the algorithm aims to effectively suppress abnormal disturbances and model errors, further enhancing positioning accuracy and robustness.

The core of the proposed fusion model is based on the following steps: first, designing an effective evaluation mechanism for GNSS positioning availability; second, achieving seamless integration between GNSS and the improved 3D PDR method. When GNSS signal quality meets positioning requirements, the system uses the position information from GNSS as the initial coordinates and fuses it with the relative displacement estimated by the improved 3D PDR based on inertial sensors. The fusion process is implemented using the RAKF, with the state and measurement equations expressed as:

(41) $$\begin{array}{l} X_{k}=A_{k-1}X_{k-1}+w_{k-1}\\ Z_{k}=H_{k}X_{k}+v_{k} \end{array}$$

where $w_{k-1}$ and $v_{k}$ are the state vectors.

Due to the complexity of pedestrian motion, it is difficult to establish an accurate functional model, and the system is susceptible to abnormal disturbances. This paper adopts an adaptive factor based on the state discrepancy statistic to resist model errors and abnormal disturbances. The adaptive factor is constructed as a two-segment function:

(42) $$\partial_{k}=\left\{\begin{array}{cc} 1, & \Delta {\tilde{X}}_{k}\leq c0\\ \frac{c0}{\Delta {\tilde{X}}_{k}}, & \Delta {\tilde{X}}_{k}>c0 \end{array}\right.$$

where c0 is an adjustable constant; $\Delta {\tilde{X}}_{k}$ is the statistic used to judge state model errors, calculated as:

(43) $$\Delta {\tilde{X}}_{k}={\left[\left\|{\tilde{X}}_{k}-{\hat{X}}_{k|k-1}\right\|/tr\left({\hat{P}}_{k|k-1}\right)\right]}^{\frac{1}{2}}$$

where tr(·) denotes the trace of a matrix, ${\tilde{X}}_{k}$ is the weighted least squares estimate of the state:

(44) $${\tilde{X}}_{k}={\left(A_{k}^{T}P_{k}A_{k}\right)}^{-1}A_{k}^{T}P_{k}Z_{k}$$

where $P_{k}$ is the weight matrix.

The gain matrix is calculated as:

(45) $$K_{k}=\frac{1}{\partial_{k}}P_{k|k-1}H_{k}^{T}{\left(\frac{1}{\partial_{k}}H_{k}P_{k|k-1}H_{k}^{T}+{\bar{R}}_{k}\right)}^{-1}$$

where $\partial_{k}$ is the adaptive factor and ${\bar{R}}_{k}$ is the equivalent weight matrix used to control observation outliers.

This paper employs the Huber method to construct the equivalent weight matrix. Its diagonal elements and off-diagonal elements are calculated as follows:

(46) $${\bar{r}}_{k_{ii}}=\left\{\begin{array}{cc} \frac{1}{\sigma_{ii}}, & \left|r_{k_{i}}^{\prime }\right|\leq c\\ \frac{c}{\left|r_{k_{i}}^{\prime }\right|}\cdot \frac{1}{\sigma_{ii}}, & \left|r_{k_{i}}^{\prime }\right|>c \end{array}\right.$$

(47) $${\bar{r}}_{k_{ij}}=\left\{\begin{array}{cc} \frac{1}{\sigma_{ij}}, & \left|r_{k_{i}}^{\prime }\right|\leq c\;and\left|r_{k_{j}}^{\prime }\right|\leq c\\ \frac{c}{\max\left\{\left|r_{k_{i}}^{\prime }\right|,\left|r_{k_{j}}^{\prime }\right|\right\}}\cdot \frac{1}{\sigma_{i,j}}, & \left|r_{k_{i}}^{\prime }\right|>c\;or\;\left|r_{k_{j}}^{\prime }\right|>c \end{array}\right.$$

where $\sigma_{ii}$ and $\sigma_{ij}$ are the diagonal and off-diagonal elements of the measurement noise covariance matrix, respectively. *c* is a constant (typically between 1.3 and 2.0). $r_{k_{i}}^{\prime }$ is the standardized residual:

(48) $$\left|r_{k_{i}}^{\prime }\right|=\left|\frac{r_{k_{i}}}{\sigma_{r_{k_{i}}}}\right|$$

where $r_{k_{i}}$ is the observation residual, and $\sigma_{r_{k_{i}}}$ is its mean deviation.

## 3. Results

### 3.1. Test Platform

**Figure 2:**
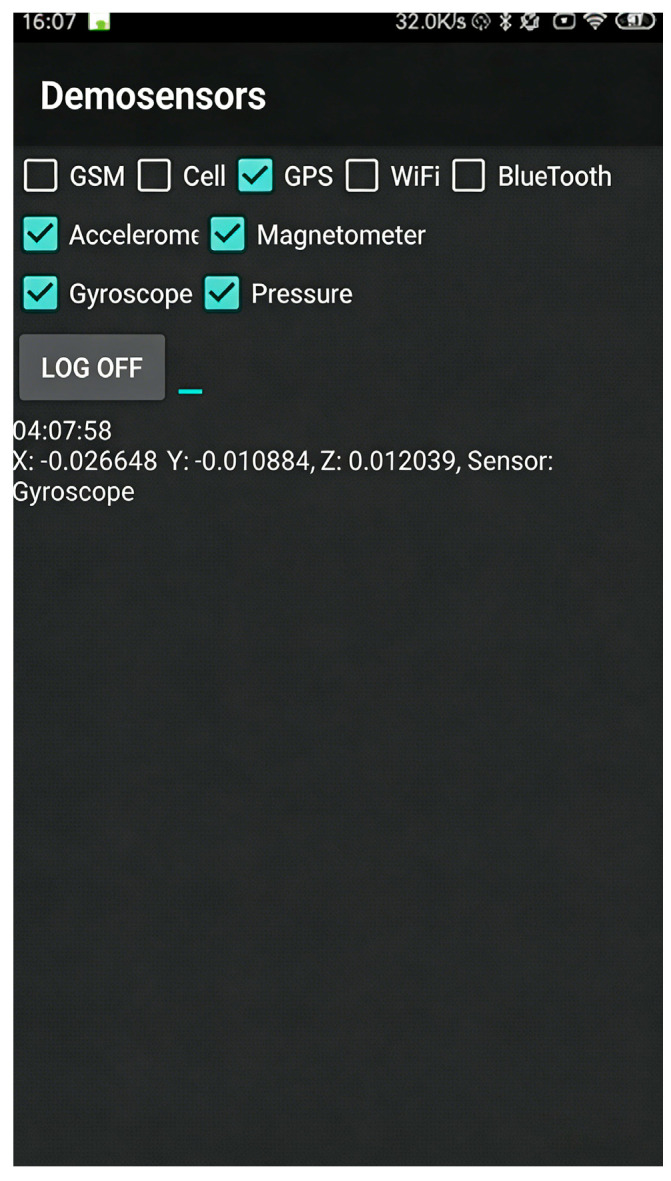
Software interface.

To evaluate the performance of the proposed method, we conducted a series of field experiments in an outdoor environment. A Xiaomi 5 smartphone was selected as the primary device. It features a Bosch BMX160 9-axis MEMS sensor (3-axis accelerometer, 3-axis gyroscope, 3-axis magnetometer) and a Bosch BMP280 barometric pressure sensor. The sensor sampling frequency was set to 50 Hz. A self-developed data collection application (Version number is v1.1) based on Android Studio was used to acquire and log sensor data in real time, with its interface shown in Figure 2. The initial state noise and measurement noise covariance matrices for the filter were determined empirically based on the collected measurements.

### 3.2. Experiment and Results Analysis in an Occluded Outdoor Environment

**Figure 3:**
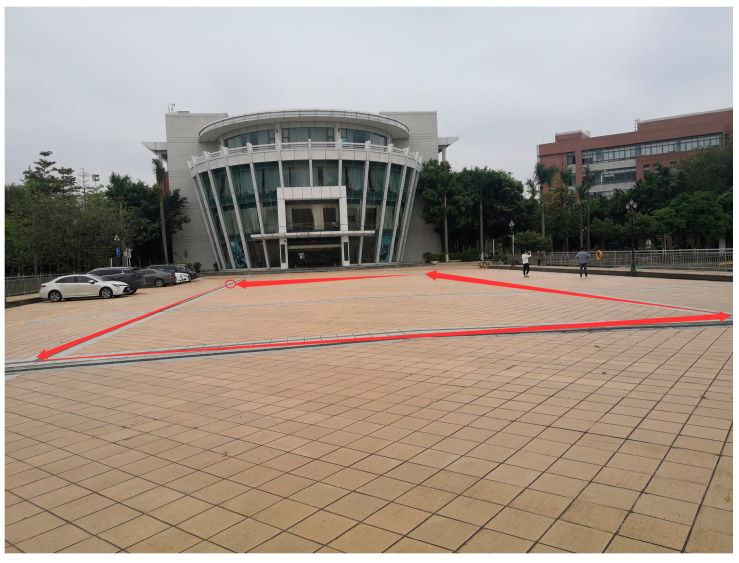
Actual view of the outdoor test site.

**Figure 4:**
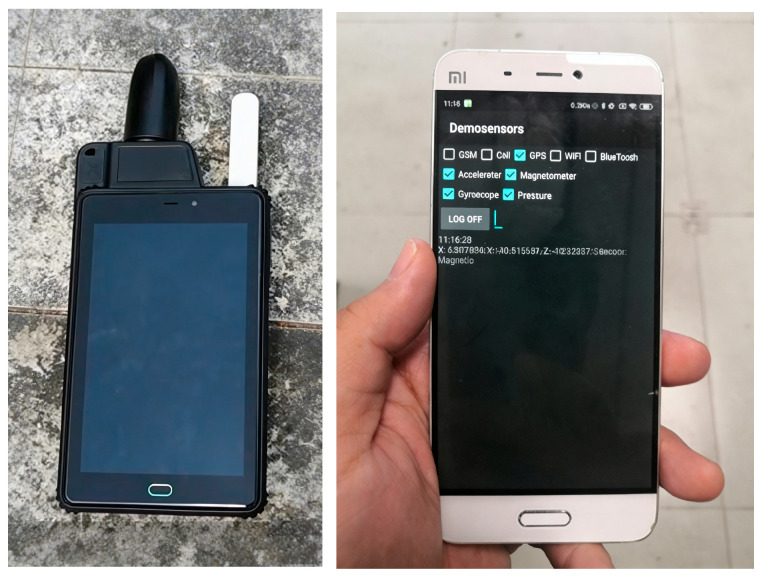
Outdoor experimental data acquisition equipment.

To simulate the complex signal conditions commonly encountered in practical applications, a partially occluded outdoor area on a university campus—surrounded by buildings and trees—was chosen as the test site. The actual view of the site is shown in Figure 3. The experimental equipment is shown in Figure 4. The experiment lasted approximately 10 min. The operator walked along a rectangular path of approximately 41 m × 21.5 m at a normal average walking speed while holding the device, eventually returning to the starting point. The smartphone was held steadily in the operator’s hand throughout the test, with its *x*-axis pointing forward in the direction of movement, the *y*-axis pointing horizontally to the right, and the *z*-axis perpendicular to the device screen, pointing upward. A high-precision RTK tablet was used simultaneously to collect position data as the ground truth reference. The MEMS sensor data were sampled at 50 Hz, the GNSS positioning output period was about 1 s, and the RTK data collection frequency was set to 1 Hz accordingly.

**Figure 5:**
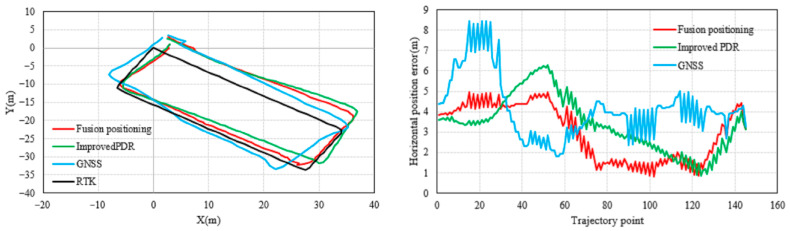
Comparison of horizontal positioning results and errors in the outdoor experiment.

The horizontal positioning results and error are shown in Figure 5, where the black trajectory represents the reference path obtained from the RTK measurements. Comparing the outputs of three methods—GNSS standalone positioning, improved 3D PDR, and the fused positioning—it can be observed that the GNSS results are most affected by the surrounding buildings and trees, showing the largest deviation. The improved 3D PDR, which incorporates adaptive and robust mechanisms to suppress error accumulation, achieves noticeably higher accuracy than GNSS. The proposed fused positioning method further integrates GNSS and PDR information via a robust adaptive Kalman filter, yielding a trajectory that best matches the reference path.

**Figure 6:**
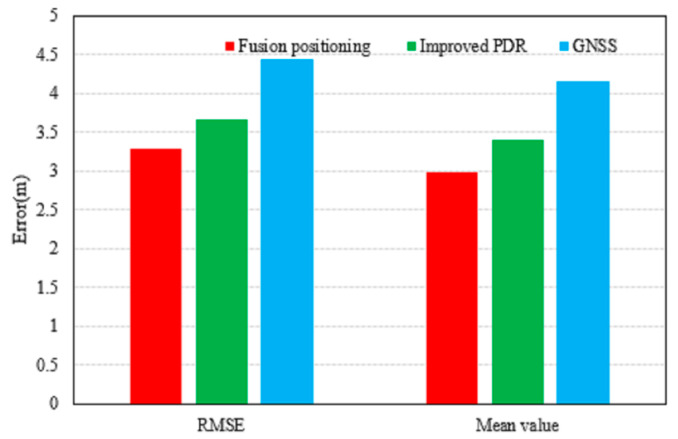
Horizontal positioning error map.

The corresponding statistical metrics provided in Table 1 and Figure 6. The fused positioning achieves a horizontal RMSE of 3.2800 m, which represents reductions of 10.39% and 25.97% compared to the improved 3D PDR (3.6606 m) and GNSS standalone positioning (4.4307 m), respectively. Moreover, its mean error is also the smallest among the three.

**Figure 7:**
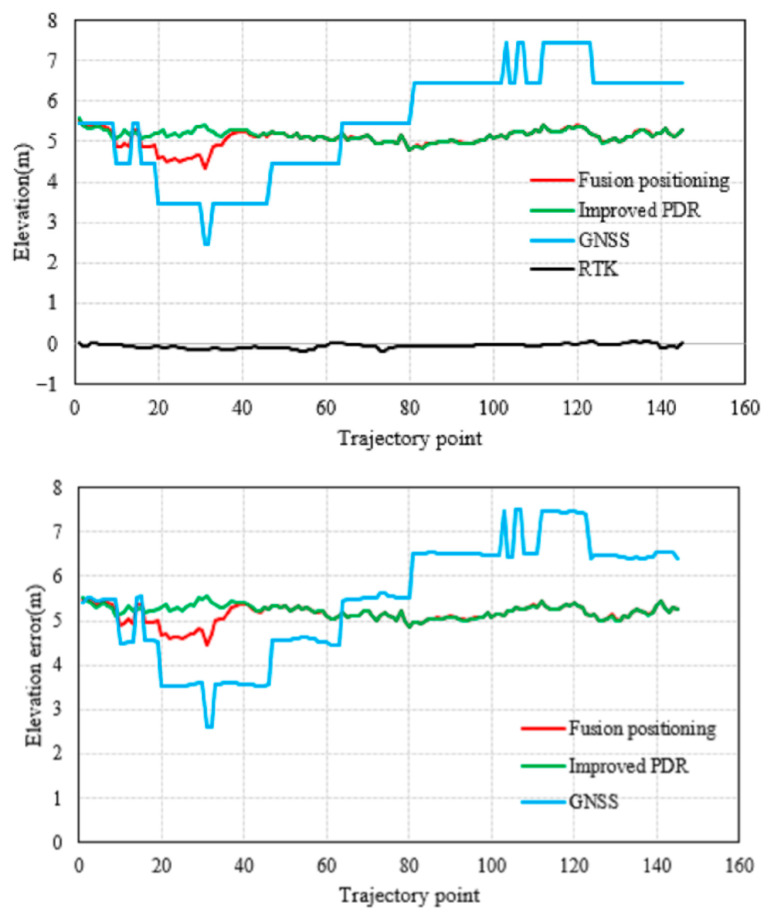
Comparison of elevation results and error in the outdoor experiment.

**Figure 8:**
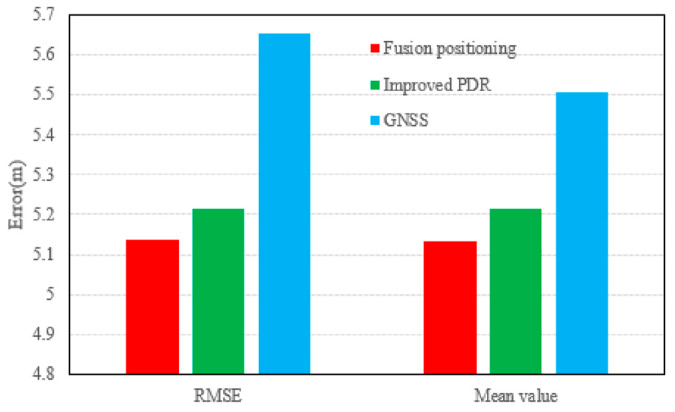
Elevation positioning error map.

The vertical positioning results and error distributions are shown in Figure 7, with statistics given in Table 2 and Figure 8. In the vertical direction, GNSS again shows the largest error due to signal occlusion. The improved 3D PDR and the fused positioning yield relatively close results. Benefiting from the filtering fusion strategy, the fused positioning still achieves a slightly better vertical RMSE (5.1378 m) compared to the PDR (5.2158 m) and GNSS (5.6524 m), corresponding to improvements of 1.50% and 9.10%, respectively.

The analysis indicates that the improved 3D PDR, as a relative positioning method, relies heavily on the accuracy of the initial coordinates. If the initial position provided by GNSS contains a large error, it will significantly affect the overall accuracy of the PDR. The proposed fusion model demonstrates substantial improvement in horizontal positioning, but the enhancement in the vertical direction is limited. This is mainly because the elevation variation in the test area is gentle, the GNSS vertical output is relatively stable, and the initial point error further diminishes the observable difference between the fused result and the individual methods.

**Figure 9:**
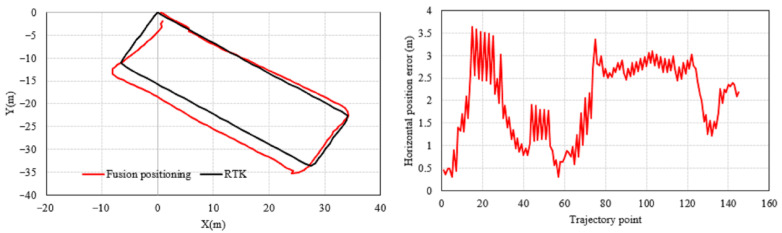
Horizontal relative positioning results and error.

**Figure 10:**
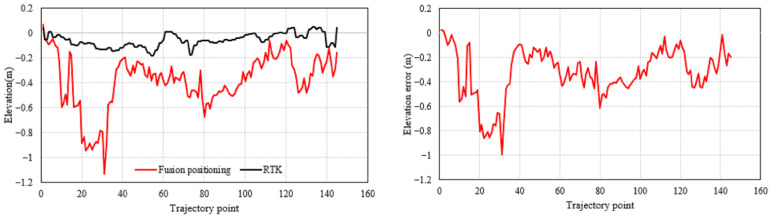
Vertical relative positioning results and error.

To further eliminate the influence of the initial point error on the evaluation, the relative positioning accuracy of the fused method was examined. The relative positioning results and errors in the horizontal and vertical directions are shown in Figure 9 and Figure 10, respectively, with corresponding statistics provided in Table 3 and Table 4. In the relative positioning scenario, the fused algorithm achieves a horizontal RMSE of 2.1921 m and a vertical RMSE of 0.3839 m; these are significantly better than the absolute positioning results. This confirms that the final accuracy of the fusion model depends not only on the filtering algorithm itself but also on the accuracy of the starting point. Therefore, how to quickly obtain a high-precision initial position without relying on external infrastructure remains a key issue worthy of further research.

## 4. Discussion

This paper proposes a tightly coupled GNSS/PDR positioning method based on smartphones. It dynamically adjusts the noise statistics of the filtering process through a robust adaptive strategy and adopts a hierarchical architecture to fuse heterogeneous sensor information. Experiments demonstrate that compared to filters such as EKF and UKF, which rely on fixed noise models or local linearization approximations, the fusion method exhibits superior state estimation stability when GNSS signals undergo drastic changes. This advantage stems from its ability to suppress model mismatch and gross error observations, aligning with assertions in adaptive filtering theory regarding enhanced estimation robustness in non-Gaussian environments. However, the effectiveness of this mechanism is constrained by the convergence speed and accuracy of the adaptive adjustment. When the signal environment experiences extreme, rapid, or atypically complex variations, the system’s statistical judgment of “state discrepancy” or “observation residuals” may suffer from lag or deviation. This can cause the adjustment of the adaptive factor and robust weight matrix to temporarily fail to keep pace with dynamic changes, leading to short-term degradation in positioning performance, thereby revealing the theoretical sensitivity and engineering vulnerability of the method in ultra-fast-changing environments.

By constructing a nonlinear step-length model and introducing joint constraints from differential barometric pressure and step frequency, this study achieves decoupled estimation of pedestrian motion in three-dimensional space. Experimental data confirm the accuracy improvement of the method in the elevation direction, demonstrating the practical value of commercial-grade barometric pressure sensors as an auxiliary information source for vertical orientation. However, the reliability of this accuracy enhancement highly depends on the quality of barometric pressure measurement data. The built-in barometric pressure sensors in smartphones are susceptible to various non-altitude factors, including (but not limited to) temperature drift caused by device self-heating, instantaneous fluctuations induced by environmental airflow (wind pressure), and the effects of changes in temperature and humidity. Although this study employs a differential barometric pressure strategy to offset some common errors, in scenarios where the barometric physical environment itself is unstable or exhibits steep gradients, the calculated elevation estimates may still exhibit significant jumps or slow drifts. This indicates an inherent bottleneck in relying solely on barometric pressure sensors for robust elevation positioning; their reliability is constrained by environmental physical conditions. Further integration with other vertical reference information possessing complementary characteristics is needed in the future.

From a theoretical framework perspective, this study constructs a lightweight solution that does not rely on pre-collected fingerprint databases or high-precision physical maps, which holds positive significance for promoting the implementation of Ubiquitous Positioning. However, examining the current scheme from the viewpoints of systems engineering and practical application reveals several limitations that must be earnestly addressed:

In extreme scenarios where GNSS signals are denied over the long term and completely, the system becomes entirely dependent on the PDR module for dead reckoning. Although this study incorporates multiple improvements in the PDR algorithm to inhibit error growth, the heading drift caused by the inherent error characteristics of low-cost MEMS inertial sensors (such as gyroscope bias instability and random walk), coupled with the uncertainty of the step length model, means that positioning errors will still diverge over time due to accumulation. The proposed method has not fundamentally solved the core challenge of maintaining bounded positioning error under conditions of prolonged absence of absolute positional reference.

The adopted hierarchical filtering (two-step estimation) architecture, while conceptually clear and capable of enhancing fusion performance, incurs the cost of introducing additional state maintenance and computational overhead. On resource-constrained mobile platforms like smartphones, continuously running this algorithm imposes a significant load on the device’s processing units (CPU/GPU), directly translating into higher energy consumption and consequently affecting battery life. This constitutes a key limiting factor for its practical application in scenarios requiring always-on background services providing continuous positioning.

The experimental validation in this study was completed using a single device model in a specific, controlled outdoor occlusion scenario. The actual performance of the algorithm may be widely influenced by numerous variables, including performance differences among sensors in various smartphone models, the diversity of device carrying positions and postures, the uniqueness of individual user gait and movement patterns, and complex electromagnetic and physical interference in broader geographical and climatic environments. The current model does not yet embed systematic adaptive or calibration mechanisms for these variables. Therefore, the generalizability and robustness of its conclusions in grander real-world scenarios still require rigorous assessment through large-scale, long-term cross-device, cross-user, and cross-region field tests.

## 5. Conclusions

This paper addresses the challenge of outdoor pedestrian positioning by proposing a novel fusion model that integrates Global Navigation Satellite System (GNSS) positioning with an improved three-dimensional pedestrian dead reckoning (3D PDR) method. The core of this model lies in the application of a Robust Adaptive Cubature Kalman Filter (RACKF), which facilitates seamless data fusion and enhances system robustness.

The proposed smartphone-based, tightly coupled GNSS/PDR positioning method employs a sophisticated two-step filtering architecture. In the first step, a quaternion-based RACKF optimizes heading estimation by effectively fusing the short-term accuracy of the gyroscope with the long-term stability of the magnetometer, thereby suppressing heading drift caused by random device orientations. The second step executes the core fusion of GNSS observations with the enhanced PDR solution. In this phase, an adaptive factor based on prediction residuals and a robust factor derived from maximum likelihood estimation are introduced. This dual mechanism dynamically mitigates GNSS multipath interference in urban canyons and compensates for time-varying noise in Micro-ElectroMechanical Systems (MEMS) sensors, significantly improving positioning continuity and resilience against outliers. Furthermore, the model incorporates a nonlinear step length model and imposes joint constraints using differential barometric height measurement and step frequency detection, enabling robust and synchronized estimation in both horizontal and vertical dimensions.

Experimental results confirm the effectiveness of this method. Compared to using GNSS or the improved 3D PDR individually, the fusion positioning model demonstrates a significant improvement in accuracy. This validates the model’s capability to provide a reliable, lightweight solution for integrated outdoor positioning, operating entirely on built-in smartphone sensors without reliance on pre-existing maps or external infrastructure.

However, this study also identifies certain limitations. The system’s long-term autonomous operation capability in extreme GNSS-denied environments remains constrained by the inherent error accumulation of the PDR component. Additionally, the computational load associated with the two-step filtering architecture imposes a non-negligible impact on smartphone power consumption. Therefore, future work will focus on optimizing the computational efficiency of the RACKF algorithm for real-time execution on mobile platforms to reduce power consumption. Moreover, we will validate the system’s generalizability through large-scale, long-term tests across diverse smartphone models, user gait patterns, and challenging urban environments to build a more robust and fully autonomous positioning system.

## Figures and Tables

**Table 1:** Horizontal positioning error statistics (m).

Type of Error	Fused Positioning	Improved 3D PDR	GNSS Standalone
RMSE	3.2800	3.6606	4.4307
Mean Error	2.9772	3.3986	4.1587

**Table 2:** Elevation error statistics (m).

Type of Error	Fused Positioning	Improved 3D PDR	GNSS Standalone
RMSE	5.1378	5.2158	5.6524
Mean Error	5.1338	5.2140	5.5038

**Table 3:** Horizontal relative positioning error statistics (m).

Type of Error	Fused Positioning
RMSE	2.1921
Mean Error	2.0132

**Table 4:** Vertical relative positioning error statistics (m).

Type of Error	Fused Positioning
RMSE	0.3839
Mean Error	−0.3262

## Data Availability

The data presented in this study are available from the corresponding author on reasonable request.

## Abbreviations

The following abbreviations are used in this manuscript:

LBS	location-based services
MEMS	micro-electro-mechanical systems
PDR	pedestrian dead reckoning
RACKF	robust adaptive cubature Kalman filter
RMSE	Root Mean Squared Error
GNSS	Global Navigation Satellite System
CF	complementary filter
KF	Kalman filter
EKF	Extended Kalman filter
UKF	unscented Kalman filter
CKF	cubature Kalman filter
ZUPT	Zero Velocity
